# Testing Independent Component Patterns by Inter-Subject or Inter-Session Consistency

**DOI:** 10.3389/fnhum.2013.00094

**Published:** 2013-03-22

**Authors:** Aapo Hyvärinen, Pavan Ramkumar

**Affiliations:** ^1^Department of Computer Science and Helsinki Institute for Information Technology, University of HelsinkiHelsinki, Finland; ^2^Department of Mathematics and Statistics, University of HelsinkiHelsinki, Finland; ^3^Brain Research Unit, O. V. Lounasmaa Laboratory, Advanced Magnetic Imaging Centre, Aalto UniversityEspoo, Finland

**Keywords:** independent component analysis, inter-subject consistency, resting-state fMRI, significance testing, group analysis

## Abstract

Independent component analysis (ICA) is increasingly used to analyze patterns of spontaneous activity in brain imaging. However, there are hardly any methods for answering the fundamental question: are the obtained components statistically significant? Most methods considering the significance of components either consider group-differences or use arbitrary thresholds with weak statistical justification. In previous work, we proposed a statistically principled method for testing if the coefficients in the mixing matrix are similar in different subjects or sessions. In many applications of ICA, however, we would like to test the reliability of the independent components themselves and not the mixing coefficients. Here, we develop a test for such an inter-subject consistency by extending our previous theory. The test is applicable, for example, to the spatial activity patterns obtained by spatial ICA in resting-state fMRI. We further improve both this and the previously proposed testing method by introducing a new way of correcting for multiple testing, new variants of the clustering method, and a computational approximation which greatly reduces the memory and computation required.

## 1 Introduction

After estimating the parameters of any statistical model, it would be reasonable to test them in some way for statistical significance, also called reliability in some contexts. In the case of independent component analysis (ICA), methods for such testing have not been widely used, nor do many exist in the first place. Methods for group-difference testing (Calhoun et al., 2009) are widely used, but the fundamental question of which components are reliable in a single group or even a single subject is rarely considered using principled statistical testing methods.

In previous work, we proposed a framework which develops such testing methods based on the concept of inter-subject consistency. The basic idea is to perform ICA separately for each subject, and define that an estimated component can be considered significant if it appears in sufficiently similar form in more than one subject (Hyvärinen, 2011). A rigorous formula for what is “sufficiently” similar was derived based on the definition of a null hypothesis and application of statistical testing theory. This provided a quantitative theoretical basis for the self-organizing group ICA method originally proposed by Esposito et al. (2005). Thus, the testing method provided, at the same time, a solution to the problem of how to do ICA simultaneously on data from many subjects, or in general, many data matrices (Calhoun et al., 2001, 2009). In fact, data from a single subject can also be tested by doing ICA separately for data from several sessions recorded from the same subject, and considering similarities between the sessions in the same way.

However, the theory by Hyvärinen (2011) was only developed for the case where the inter-subject consistency was seen in the columns of the mixing matrices. This is relevant in particular to the case of temporal ICA, typically applied on EEG and MEG, where the mixing matrix gives the spatial patterns of activity. Yet, the most common application of ICA in brain imaging is the spatial ICA of fMRI data, often measured at rest (Kiviniemi et al., 2003; van de Ven et al., 2004; Beckmann et al., 2005). A related spatial ICA method was recently proposed on MEG as well by Ramkumar et al. (2012). For such spatial ICA, inter-subject consistency is usually measured between the spatial patterns which are the independent components themselves, and not the columns of the mixing matrix.

Here, we adapt the theory by Hyvärinen (2011) for the case where the inter-subject consistency is sought among the independent components, as in spatial ICA of resting-state fMRI. We propose a generalization of the null hypothesis by Hyvärinen (2011) to accommodate the case of testing the independent components. We take an empirical approach to modeling the null distributions since a purely analytical approach like in Hyvärinen (2011) does not seem feasible. We also propose a number of improvements and generalizations to the general framework, which can be used in the case of testing the mixing matrix as well. Like the method in Hyvärinen (2011), the current method can be directly applied on data from different recording sessions of the same subject as well.

## 2 Mathematical Theory

### 2.1 Clustering of components by inter-subject consistency

Assume we have measurements of *r* subjects or sessions. Denote by **X***_k_*, *k* = 1, …, *r* the data matrix for the *k*-th subject or session. For simplicity of terminology, we assume in the following that the data comes from different subjects and not sessions. If the data matrix comes from fMRI recordings, and we are to perform spatial ICA, each row is one time point (one volume) and each column a voxel. Assume we have performed ICA separately for all the subjects, obtaining the estimated decompositions.

(1) $${\hat{S}}_{k}={\hat{W}}_{k}X_{k},\text{ or }X_{k}={\hat{A}}_{k}{\hat{S}}_{k},$$

where ${\hat{A}}_{k}$ is the pseudoinverse of ${\hat{W}}_{k}$. In the following we only analyze ${\hat{S}}_{k}$, so it is immaterial whether any dimension reduction is done by PCA, and whether the ${\hat{W}}_{k}$ and ${\hat{A}}_{k}$ are in the whitened space or in the original.

Now, following Esposito et al. (2005), we want to combine the ICA results for the different subjects by clustering. That is, we try to find components which are similar enough in different subjects, so that we can consider them to correspond to the same underlying component. Each such cluster of sufficiently similar components (i.e., components with sufficient inter-subject consistency) is then considered a single group-level component in subsequent analysis. The key challenges in such a method are to find principled and practical definitions for similarity, and to define the thresholds regarding when the components are similar enough to be considered the same.

Our goal here is to devise a statistical test to determine if some of the rows of ${\hat{S}}_{k}$ are sufficiently similar for different *k* in the sense that the similarity cannot be due to chance. We assume here that the rows of ${\hat{S}}_{k}$ model the phenomena of interest (e.g., spatial patterns of brain activity in fMRI) whose inter-subject consistency we want to test. In contrast, we do not assume that the **A***_k_* have any inter-subject consistency. For example, in spatial ICA of fMRI, the **A***_k_* give the time courses which hardly have any inter-subject consistency in the case of resting-state activity.

The key to a principled statistical test is the definition of a null hypothesis, H_0_. The null hypothesis should model the case where the ICA results for different subjects are completely independent of each other in the sense that the components in different subjects have no similarity at all, other than what would be expected by chance. As argued by Hyvärinen (2011), the randomness can in fact come from two different sources:

1. It could be that the ICA algorithm fails completely, or
2. It could be that the underlying data are completely different for each subject in the sense that the brain networks are completely different from each other.

We will begin by introducing a null distribution which embodies these two sources of randomness.

### 2.2 Definition of null distribution

In order to model the randomness in the ICA estimation procedure, we define a null hypothesis as follows. We assume, following Hyvärinen (2011), that the estimated ${\hat{A}}_{k}$ are random orthogonal transformations of the actual mixing matrices. Denote by **U***_k_* random orthogonal matrices (more precisely, matrices uniformly distributed in the set of orthogonal matrices). Under the null hypothesis we have, for the estimated decompositions:

(2) $${\hat{A}}_{k}=A_{k}U_{k}\text{ or }{\hat{W}}_{k}={\text{U}}_{k}^{T}W_{k}$$

where **A***_k_* and **S***_k_* below denote the actual underlying values of those parameters or random variables, as opposed to the estimates ${\hat{\text{A}}}_{k}$ and ${\hat{\text{S}}}_{k}.$ This randomness due to the **U***_k_* models errors in the ICA estimation procedure. The idea is to assume that the prewhitening step in ICA was successfully performed, but the ICA algorithm returned a random result, i.e., a random orthogonal transformation in the whitened space. This is equivalent to assuming that the estimates of the **S***_k_* are random orthogonal rotations of the actual **S***_k_*:

(3) $${\hat{\text{S}}}_{k}=U_{k}^{T}S_{k}$$

since $X_{k}={\hat{\text{A}}}_{k}{\hat{\text{S}}}_{k}=A_{k}S_{k}.$

We have to further model randomness in the actual independent components, due to individual differences in brain anatomy and physiology. In our previous model (Hyvärinen, 2011), the randomness relating to the actual individual differences of the brains was assumed to be reflected in this same orthogonal rotation, since the spatial patterns corresponded to the columns of **A***_k_*. This assumption was justified in the case of testing the mixing matrix, e.g., in the case of temporal ICA of EEG or MEG. However, when testing for similarities of the independent components, that assumption does not seem to be adequate. This is because if the individual differences of the brains were modeled by a random rotation of the spatial patterns as in equation (3), we would be violating the ICA model, since such a random rotation would make the components dependent. Therefore, we need to model the individual variability of the brains by a separate random model. The random model should give random spatial patterns which still follow the ICA model, i.e., are independent for each subject.

The approach we take here is to assume that under the null hypothesis H_0_, the rows of the **S***_k_*, denoted by **S***_ki_*, follow the same multivariate distribution *p_s_*(**S***_ki_*). In general, this is a stochastic (spatial) process which models the hypothetical generation of spatial patterns given by the independent components. Drawing each **S***_ki_* randomly and independently of each other from *p_s_* does give us a number of components which are, by construction, independent, and thus respect the assumptions of the ICA model.

In the case of spatial ICA, the distribution *p_s_* essentially models the spatial regularities of the patterns, including patterns of brain activity or artifacts on the one hand, and measurement noise on the other. We cannot assume, for example, that the voxels are all independent of each other, since this would grossly overestimate the degree of randomness, and thus underestimate the similarities obtained by chance.

Here, we do not attempt to construct an explicit model of *p_s_*. Instead, we construct an empirical model of the null distribution of the similarities between the components, which is the relevant quantity for the construction of tests, as will be discussed next.

### 2.3 Empirical model of null distribution of similarities

We define the similarities of the components of two subjects k≠1 as the entries of the following matrix:

(4) $$\Gamma_{kl}={\hat{\text{S}}}_{k}{\hat{\text{S}}}_{l}^{T}$$

This simple definition assumes that the estimated rows ${\hat{S}}_{k}$ are zero mean, and constrained to unit norm. The ${\hat{S}}_{ki}$ are further assumed orthogonal for each subject, i.e., for *i* ≠ *j* for fixed *k*. For example, components estimated by FastICA always fulfill the orthogonality and norm constraint after the means have been subtracted from the estimated components.

The central problem is how to model the distribution of the matrix Γ under the null hypothesis. For simplicity, we only attempt to model the marginal distributions of the entries in this matrix and approximate the joint distribution by assuming independence of the entries. Denote this marginal distribution by *p*_γ_. We take here an empirical approach and fit a parametric model to the statistics of the measured similarities to model *p*_γ_.

Under H_0_, we have

(5) $$\Gamma_{kl}=U_{k}^{T}S_{k}S_{l}^{T}U_{l}^{T}$$

where **U***_k_* and **U***_l_* are random orthogonal matrices independent of each other, and the rows of **S***_k_* and **S***_l_* are obtained from the prior distribution *p_s_*. It is, in fact, possible to obtain an empirical sample of *p*_γ_ by the following procedure: take the matrices of the estimated independent components ${\hat{\text{S}}}_{k}$, make a number of random rotations as $V_{k}{\hat{\text{S}}}_{k}$, and compute the similarities

(6) $${\tilde{\Gamma }}_{kl}=V_{k}{\hat{S}}_{k}{\hat{S}}_{l}^{T}V_{l}^{T}.$$

This has the distribution of ${\bar{U}}_{k}S_{k}S_{l}{\bar{U}}_{l}$ where ${\bar{U}}_{k}=V_{k}U_{k}^{T}$ is again a random orthogonal matrix (and likewise for the index *l*). Thus, the constructed matrix follows the same distribution as the similarity matrix ${\tilde{\Gamma }}_{kl}$ under H_0_. In principle, we could obtain a Monte Carlo sample of this distribution by generating random orthogonal matrices, but we will show next that this is not necessary.

It was pointed out by Hyvärinen (2011) that the distribution of the square of each entry of $U_{k}^{T}U_{l}$ follows a beta distribution Beta(*α*, *β*) with parameters *α* = 1/2 and *β* = (*n* − 1)/2 where *n* is the dimension of the data **X***_k_* (after a possible dimension reduction by PCA). So, we decide to fit a Beta(1/2, *β*) distribution to the entries of the random matrix ${\tilde{\Gamma }}_{kl}$, with *β* being the free parameter. This should provide a reasonable approximation, and as we will see next, this approximation leads to a particularly simple method.

A basic way of estimating the parameters in a beta distribution is given by the moment method. A well-known formula gives the expectation of a beta-distributed random variable *u*^2^ as

(7) $$E\left\{u^{2}\right\}=\frac{\alpha }{\alpha +\beta }$$

from which we can derive, using the method of moments, the estimator of *β* with known *α* = 1/2 as

(8) $$\hat{\beta }=\alpha \left({\left[E\left\{u^{2}\right\}\right]}^{-1}-1\right)=\frac{1}{2}\left({\left[E\left\{u^{2}\right\}\right]}^{-1}-1\right)$$

Thus, we see that parameter *β* can be estimated based on the expectation of the squares of the matrix of similarities after random rotations.

Using the expectation of squares leads to a dramatic simplification of the method. Since the expectation of squares is taken over all the elements of the matrix, we can think of it being first taken over all the elements of the similarity matrix for each subject pair $\Gamma_{kl}$, and then over different subject pairs *k*, *l*, *k* ≠ *l*. Now, the orthogonal transformations in equation (6) do not change the sum of the squares of the elements of the matrix, so they can be omitted. Thus, we do not need to take the random rotations into account in the estimation of *β*, and no Monte Carlo simulation of the distribution is necessary. We can simply estimate *β* using the sum of squares of the computed similarity matrices $\Gamma_{kl}$ as

(9) $$\hat{\beta }=\frac{1}{2}\left(\tilde{n}-1\right)$$

with

(10) $$\tilde{n}={\left[E\left\{\gamma^{2}\right\}\right]}^{-1}=\frac{n^{2}r\left(r-1\right)}{\underset{ij,k\neq l}{\sum }\gamma_{kl,ij}^{2}}$$

where $\gamma_{kl,ij}^{2}$ is the *i*, *j*-th entry in the matrix $\Gamma_{kl}$. Here, the quantity *ñ* can be considered as measure of the “randomness,” i.e., lack of structure, of the independent components. If the independent components are very random in the sense of having no spatial structure (e.g., white noise), the similarities in the denominator will be small and this quantity will be large; however, *ñ* depends on the data dimension as well. In fact, *ñ* coincides with a parameter which gives the “effective” data dimension in the original framework by Hyvärinen (2011).

Thus, to test the hypothesis, we only need to estimate *β* as $\hat{\beta }$ in equation (9) and then compute the p-values based on the beta distribution.

### 2.4 New corrections for multiple testing

The p-values for the connections (similarities) computed above can be used in a hierarchical clustering procedure to create clusters which contain one component from as many subjects as possible using only significant connections. Since we will be testing many possible candidates to be included in the clusters, we need some corrections for multiple testing.

As proposed by Hyvärinen (2011), we control here the false positive rate (FPR) for the formation of clusters, and the false discovery rate (FDR) for adding new elements to clusters. This is because claiming the existence of a cluster which does not actually exist can be considered a more serious error than adding an extra component to the cluster, and thus we want to be more conservative in forming new clusters.

For controlling the number of falsely formed clusters, we thus use Bonferroni correction like in Hyvärinen (2011). Denoting by *α*_FP_ the uncorrected false positive level, we obtain the corrected level as

(11) $$\alpha_{\text{FP}}^{\text{corr}}=\frac{\alpha_{\text{FP}}}{m}$$

where the number of tests is

(12) $$m=\frac{nr\left(r-1\right)}{2}$$

with *r*, the number of subjects, and *n*, the dimension of the data. The goal here is to make the probability of inferring even one wrong cluster smaller than *α*_FP_. This is essentially the same as the family wise error rate.

Regarding the process of adding further components to the cluster after it has been formed, we develop here a method related to FDR. The problem with using ordinary FDR as in Hyvärinen (2011) is that in computing the true and false positives, it uses the number of connections which are considered true, while we are interested in the number of components which are added to the clusters. To see why these may not be closely related, consider a true cluster of 10 components. It contains 45 connections within itself, and thus the number of true connections within the cluster should be taken as 45. Now, if we falsely infer one of the outgoing connections to be true, we would calculate the FDR to be 1/46. However, since this means that we will have 11 components, one of which is falsely added to the cluster, it would make more sense to say we have an FDR of 1/11. The relationship between the FDR of connections and the FDR of components is thus quite complicated.

Since it is not straightforward to define the number of false discoveries in this problem, it is not clear how generic FDR methods, such as Simes’ procedure (Simes, 1986; Benjamini and Hochberg, 1995) should be applied, as already pointed out by Hyvärinen (2011). Next, we provide one possible definition of false discoveries (and their rate) which attempts to optimally adapt the concept to the problem at hand. The number of false discoveries is basically the number of components falsely added to any of the clusters.

Consider a cluster which actually has *c* components, all of them true ones. There are *c*(*r* − *c*) connections which go out of that cluster (we are considering maximal connections only as explained below in Section 2.5), each of which can give rise to a false positive. Given a corrected *α*${\text{α}}_{\text{FD}}^{\text{corr}}$ level used in the test, and considering the tests independent, we would have an FDR which is smaller than

(13) $$\frac{\alpha_{\text{FD}}^{\text{corr}}c\left(r-c\right)}{c}=\left(r-c\right)\alpha_{\text{FD}}^{\text{corr}}$$

where we omit the false positives in the denominator to obtain a simple upper bound. To guarantee that this is smaller than a given FDR rate *α*_FD_, we can simply choose

(14) $$\alpha_{\text{FD}}^{\text{corr}}=\frac{\alpha_{\text{FD}}}{r-2}$$

which makes (13) less than or equal to *α*_FD_ for any *c* > 2 (in the case *r* = 2, i.e., only two subjects, the FDR is not used anyway). Thus, we propose to use the correction in equation (14) in the testing. It controls the FDR in the sense of the number of falsely added components.

### 2.5 Computational simplification

Next, we propose to reduce the computational resources (both memory and CPU time) by a simple approximation. After computing the similarities of the components of two subjects in matrix $\Gamma_{kl}$, we only store the maximum similarities of each component with the components of the other subject. In other words, we only store the maxima of the rows and columns of $\Gamma_{kl}$, as well as the indices obtaining those maxima. This is justified because a component can belong to only one cluster anyway, and it is most likely to be the one with the most significant similarity.

This reduces the amount of memory needed by a factor of *n/*2, and the computation time is reduced by a similar amount although its exact computation is not straightforward. Hyvärinen (2011) found that the computational bottleneck of the method is in the memory needed for storing all the similarities, so this reduction in memory storage is what perhaps most matters in practice.

We need to find the distribution for these maxima. We propose a simple approximation assuming the elements of the similarity matrix are independent, and by applying basic probability calculus, which gives

(15) $$P\left(\underset{i}{\max}\;s_{i}\leq \alpha \right)=\overset{n}{\underset{i=1}{\prod }}P\left(s_{i}\leq \alpha \right)$$

for independent variables *s_i_*.

### 2.6 Different clustering strategies

We further propose that the clustering can use different strategies. The method proposed by Hyvärinen (2011) is related to the single-linkage strategy in hierarchical clustering, and adds a new component to a cluster by finding the largest similarity (which here means minimum p-value) among the similarities from the cluster to components not yet clustered (belonging to subjects not yet in the cluster). The classical alternatives to such a single-linkage are average-linkage and complete-linkage.

We propose to use complete-linkage as an alternative strategy in our testing method. Adapted to our specific clustering scheme, the idea is that we add a component to a cluster by considering the *maximum* of the p-values of the similarities from within the cluster to components in the remaining subjects (who do not have components in that cluster). The component with the smallest maximum of p-values will be added to the cluster. In particular, this means that a candidate component can be added to the cluster only if the connections from *all* the components inside the cluster are significant, because otherwise the maximum p-value would not be significant. (Since we store only the strongest connections between subjects, we have to also check that all the maximizing links stored point to the same component. If they don’t, the component will not be considered for inclusion.)

Using complete-linkage alleviates the well-known drawback of the single-linkage strategy, which is that when components are added one-by-one to the cluster, they can be more and more different from the two components which started the cluster. The last component to be added can be so different that the cluster cannot be considered very meaningful anymore. On the other hand, complete-linkage has the drawback of sometimes leading to conservative cluster formation.

The average-linkage strategy is often considered a useful compromise between the single-linkage and complete-linkage strategies. As an implementation of the principle of average-linkage, we further propose here a method called median-linkage. The idea is that the median of the p-values of the connections to the new component has to be significant, which means half of the connection from the cluster have to be significant^1^. This should provide an interesting compromise between single-linkage and complete-linkage.

## 3 Experimental Methods

Next, we validated the testing method proposed above by simulations and experiments on real data.

### 3.1 Simulation 1: Data resembling fMRI

As a basic test for the validity of our method, we created independent components which resemble those obtained in a (resting-state) fMRI experiment. The number of subjects was fixed to 12, and the number of independent components (or PCA dimension) was fixed to 40.

The consistent spatial patterns were small blobs in a grid. The size of the grid was 25 × 25 because this seems to be statistically the closest to real fMRI data in terms of giving a similar effective dimension *ñ*. FMRI data of course has more voxels, but the correlations between the voxels are strong, and thus the statistics of similarities are more similar to our simulations on such a small grid^2^.

**Figure 1:**
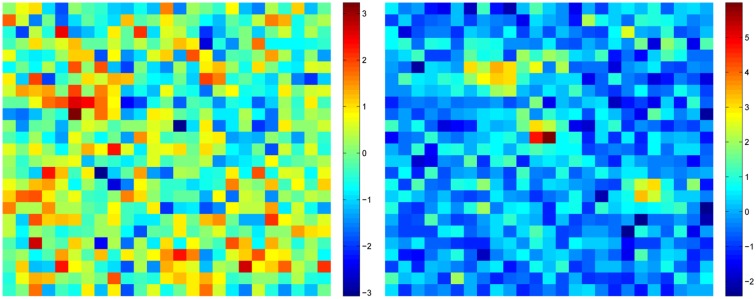
**Two patterns of activity used in Simulation 1**. Left: z-level 1.5; Right, z-level 4. Z-level means the ratio of maximum of activity blob to noise standard deviation, i.e., maximum z-score of the signal.

Various amounts of Gaussian white noise were added to the blob-like patterns to simulate measurement noise. The signal-to-noise ratio was quantified as the z-score of the activity blobs: the noise always had standard deviation equal to one, whereas the maxima of the blobs were varied in the range of 1–5, and this we called the z-level of the pattern. Some examples are shown in Figure 1.

In the basic setting, half of the subjects (the “consistent subjects”) had 20 consistent components (half of the components). In the consistent components, the underlying spatial patterns were equal for all subjects, but the measurement noises were independent for different subjects. The rest of the subjects (the “non-consistent subjects”) had patterns consisting of Laplacian white noise, generated independently of each other. Laplacian white noise is a simple model for components which are sparse and reasonably independent, thus having properties similar to blobs in fMRI data^3^. The measurement noise added to the non-consistent subjects had the same variance as the spatial patterns. After adding the noise, all the patterns were normalized to unit variance.

We created data from four different scenarios. In Scenario 1, the measurement noise was Gaussian, the single-linkage strategy was used for clustering, and as already mentioned, the proportion of consistent subjects and components was one half. We varied these basic settings one at a time to produce the other scenarios. In Scenario 2, we investigated the effect of more consistency in the data, and thus set the number of consistent subjects and consistent components to be 3/4 instead of 1/2 as in Scenario 1. In Scenario 3, we applied the complete-linkage strategy for clustering, while the data was like in Scenario 1. In Scenario 4, we investigated the effect of non-Gaussian noise: the noise was Laplacian, while other parameters were like in Scenario 1. The Laplacian distribution is not meant as a physically realistic noise model (Wink and Roerdink, 2004); its purpose is to model heavy-tailed noise possibly consisting of outliers and other deviations from the model.

For comparison, we applied the method by Hyvärinen (2011) on the same spatial patterns. While the method by Hyvärinen (2011) was not really conceived for this purpose, it is possible to input the obtained spatial patterns to that algorithm to obtain a useful baseline.

We ran 250 trials with *α*_FP_ = *α*_FD_ = 10% and computed a number of quantities to characterize the clustering results:

- the false positive rate for clusters. A cluster was considered false positive if it didn’t include the same consistent component from at least two different consistent subjects. We ignored the actual number of false positive clusters and simply computed if there was at least one such cluster for each trial. Averaging this over trials, we computed the probability of having at least one false positive cluster, which is then compared to the FPR defined above.
- the false discovery rate of further connections. First we determined for each cluster the component which was most often present among the consistent subjects. False discoveries were then defined as components which either came from the non-consistent subjects, or came from consistent subjects but were not the same component as the one most often present (if the cluster was false positive, all the components were considered false discoveries). Their number was divided by the total number of components clustered to give the FDR. We took the median of FDR over the trials since taking a mean of rates is not very meaningful.
- The number of “perfect” clusters found. As in Hyvärinen (2011), we defined a perfect cluster as one which contains the same component from all consistent subjects, and no components from the non-consistent subjects. This is basically a rather stringent measure of true positives found by the methods. The number was averaged over trials.
- Finally, we computed the total number of clusters found (including false positives), and averaged it over trials.

### 3.2 Simulation 2: New variant for testing the mixing matrix

While the theory presented in this paper is primarily intended to extend our earlier theory to testing the independent components, we have also proposed two ideas which can be used to improve the testing of the mixing matrix. In particular, our explicit FDR control formula in Section 2.4 and the computational simplification in Section 2.5 should improve the method in Hyvärinen (2011). Also, the new linkage strategy in Section 2.6 could be used as an option. To investigate this possibility, we provide here a simulation in which we use the present theory for testing the mixing matrix.

Here, we replicate Simulation 1 in Hyvärinen (2011) with the new FDR formula and the computational simplification. (We do not consider the alternative linkage strategies here.) The simulation consists of artificial data of five different scenarios in which FPR and FDR are explicitly defined, see Hyvärinen (2011) for details.

The goal of the simulation is to see if both *α*_FP_ and *α*_FD_ are still well controlled if use the introduced modification to test the mixing matrix. We set both to error rates to 0.05 in the testing method.

### 3.3 Simulation 3: Computational complexity

Next, we investigated the computational complexity of the method, using the same framework as in our earlier work (Hyvärinen, 2011).

First, to allow straightforward comparison with Hyvärinen (2011), we took the procedure of Simulation 4 from that paper without any changes, except for trying out larger dimensions. In particular, we applied the testing on the columns of the mixing matrix (which is possible as pointed out above).

Here, no ICA was done, instead we randomly generated data which models the mixing matrices obtained by ICA. We took the number of subjects to be equal to the number of independent components, using the values 8, 16, 32, 64, 128, 256, and 512 for those parameters. We generated the data so that for half of the subjects, half of the components were consistent (in fact, equal). For half of the subjects, the mixing coefficients were pure noise, and for those subjects with half consistent components, the other half of the mixing matrix was noise. The actual data generation procedure does not have a lot of influence on the computational complexity, but what is important here is that the data contains significant clusters whose number is proportional to the data dimension, and their size is proportional to the number of subjects.

We set *α*_FP_ = *α*_FD_ = 0.05. The computations were done using Matlab on a rather ordinary Linux desktop computer system with two cores of 2.66 GHz each, and 2.4 GB of memory available.

To assess the complexity, we computed the CPU time needed as well as the memory needed. The memory usage considered only the memory needed for storing the explicit variables, i.e., the final values of any Matlab operations neglecting any intermediate results, and thus clearly provides a lower bound only.

Second, we did the same simulations for testing the independent components in a more fMRI-like setting. We generated the independent component matrices **S***_k_* randomly with the same idea of half the components being consistent for half the subjects. The number of voxels (data points) was taken to be 10,000. The same settings for the number of subjects and independent components were used.

### 3.4 Experiments on real fMRI data

Finally, we applied the method on real fMRI data from Malinen et al. (2010). The data consisted of 10-min resting-state 3 T fMRI data obtained from 10 healthy subjects (37–64 years; mean 50 years; 8 males, 2 females). The statistical parametric mapping software SPM2^4^ was used to preprocess the fMRI data, including realignment, skull-stripping, normalization into the Montreal Neurological Institute (MNI) standard space, and smoothing with a 6-mm (full-width at half-maximum) Gaussian filter. For further details about fMRI data acquisition and preprocessing, see Malinen et al. (2010).

From each individual subject’s data, we reduced the dimensionality to 48 using principal component analysis (PCA) and subsequently extracted 48 spatial independent components (ICs) using FastICA (Hyvärinen, 1999). While methods have been proposed for automatically estimating the PCA dimension (Beckmann and Smith, 2004), their application is not without problems (Abou-Elseoud et al., 2010), which is why we simply fix the PCA dimension here. Our testing framework further assumes that the PCA dimension is the same for different subjects, while it would, in principle, be possible to estimate it separately for different subjects (Beckmann and Smith, 2004).

We applied the method using two different false positive rates and false discovery rates, set to either *α*_FP_ = *α*_FD_ = 0.05 or *α*_FP_ = *α*_FD_ = 0.01. In addition, we also investigated the effect of the two different linkage strategies during hierarchical clustering: single and complete-linkage. Finally, we applied the method for two further PCA dimensions, 25 and 75, where we fixed *α*_FP_ = *α*_FD_ = 0.05, and adopted the complete-linkage strategy.

## 4 Results

### 4.1 Simulation 1: Data resembling fMRI

**Figure 2:**
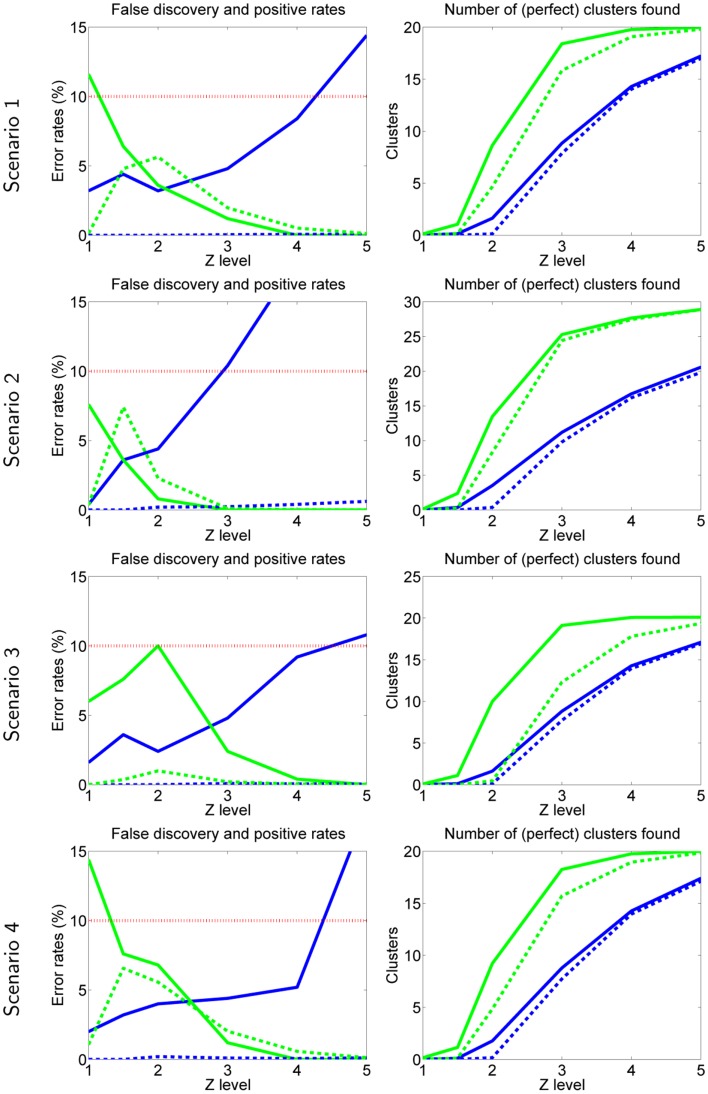
**Simulation 1: data resembling fMRI**. Each row is one scenario, briefly: scenario 1 is basic setting, scenario 2 has more inter-subject consistency, scenario 3 uses complete-linkage, scenario 4 has non-Gaussian noise. In all plots, green curves are obtained by the method proposed here, and blue curves by the method proposed by Hyvärinen (2011), given for comparison. In error rates (left), solid line is FPR and dashed line FDR. In number of clusters (right), dashed line gives the number of perfect clusters, solid line gives the total number of clusters (including false ones). The desired rates *α*_FD_ = *α*_FP_ = 0.10 are shown by the dotted red line.

The results are shown in Figure 2. Basically, our new method has quite well controlled error rates (less than the set *α*_FP_ = *α*_FD_ = 10%) in most cases (green curves on the left).

The FPR rates reach 10% in many cases, and go to 15% in the case of non-Gaussian noise (scenario 4). The case of non-Gaussian noise makes the distributions have heavier tails and therefore our method seems to slightly underestimate the probability of false positives. On the other hand, Laplacian noise is quite non-Gaussian and presumably more non-Gaussian than typical fMRI measurement noise.

The FDR are always clearly lower than the desired 10%. This may not be surprising since our corrected FDR threshold was constructed to be conservative.

On the other hand, for our previous test proposed in Hyvärinen (2011), the FPR rates are not properly controlled, and sometimes exceed 10%, while the FDR are extremely small (so close to zero that they are not clearly visible). The fact that the error rates are not controlled is not very surprising considering that the test in Hyvärinen (2011) was designed for a different kind of test. Thus, this result merely confirms that we cannot directly use our earlier theory for testing independent components themselves, and the present developments are necessary.

Furthermore, the proposed test has clearly more power than the one in Hyvärinen (2011), which is seen in that fact that it finds more perfect clusters, as well as clusters in general (right-hand side panels in Figure 2).

Overall, there is surprisingly little variation between the four different scenarios.

### 4.2 Simulation 2: New variant for testing the mixing matrix

**Figure 3:**
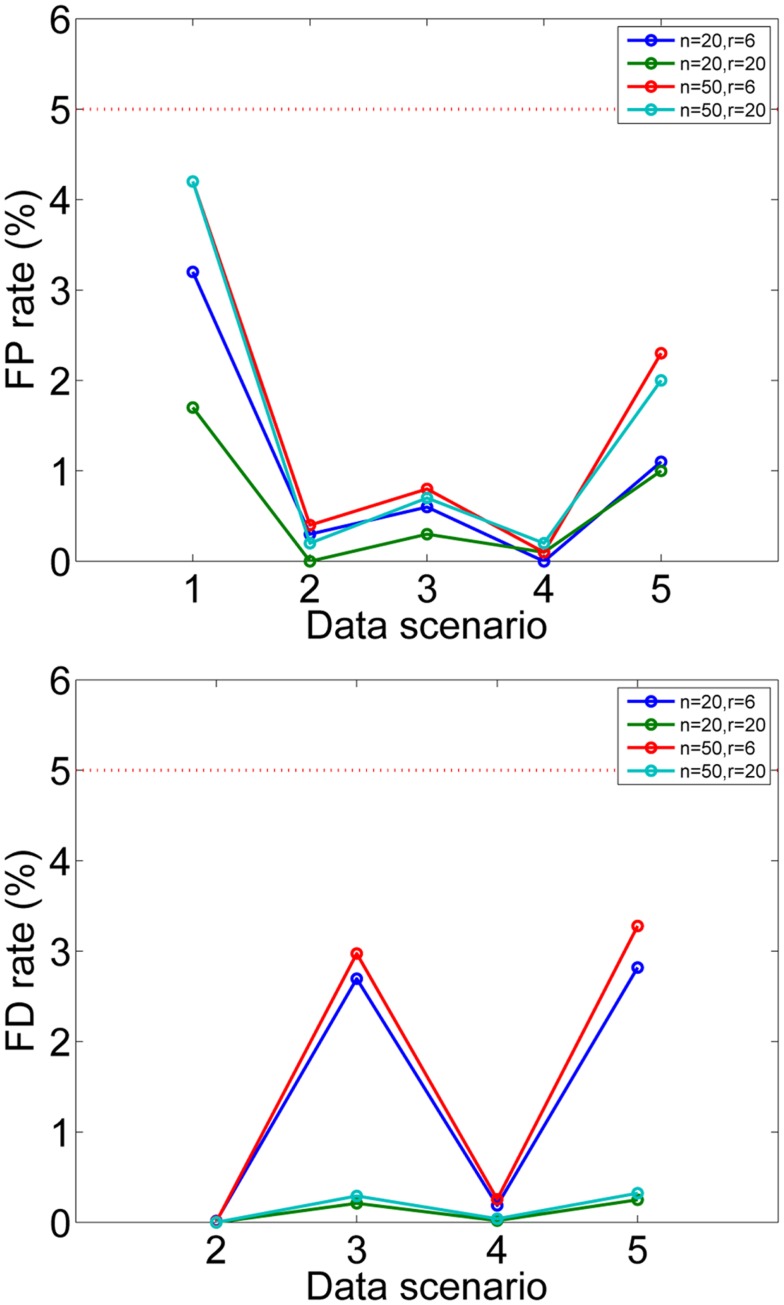
**Simulation 2: testing the mixing matrix**. False positive rates and false discovery rates are shown for simulated data. Different settings of data dimension *n* and number of subjects *r* are given in different colors. The data scenarios are explained in detail in Hyvärinen (2011), briefly: 1: no consistent components, 2: half of components consistent for all subjects, 3: all components consistent for half of the subjects, 4: for half the subjects, all components consistent and half of the components consistent for the rest of the subjects, 5: for half of the subjects, half of the components were consistent. The desired false positive and discovery rates *α*_FP_ = *α*_FD_ = 0.05 are shown by the dotted red line. For scenario 1, FDR cannot be meaningfully computed since the number of true positives is zero.

The false positive rates and false discovery rates, as defined in Hyvärinen (2011) are shown in Figure 3 for the different data-generating scenarios of Hyvärinen (2011). We can see that they are all less than the required 5%, and thus well controlled in spite of the further approximations done in developing our method in addition to the ones in Hyvärinen (2011). In fact, the approximation made in the computation of the p-values seem to lead to conservative testing, so the FPR and FDR do not need to be chosen particularly small.

### 4.3 Simulation 3: Computational complexity

**Figure 4:**
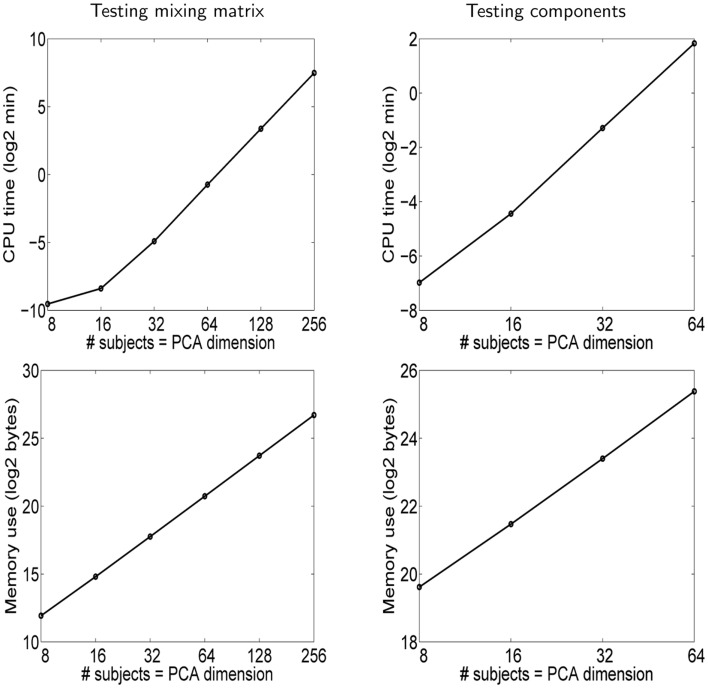
**Simulation 3: computational complexity**. The results are shown in each case as far as our computer was able to perform the computations, i.e., in the case of the mixing matrix, *n* = *r* = 512 was infeasible because it would have required more memory than was available, and likewise for *n* = *r* = 128 in the case of testing independent components.

The results are shown in Figure 4. Regarding the testing of the mixing matrix, we see a clear improvement with respect to Hyvärinen (2011). Both the memory and the CPU time needed are decreased approximately by a factor of 20 for the largest data set^5^. Thus, the optimized method greatly expands the applicability of the method, for example to the case of databases with hundreds of subjects.

In the case of testing the independent components, both the memory and the CPU time requirements are larger than in the case of testing the mixing matrix, approximately by a factor of five in the case of the largest data set. This is understandable since the independent components have much larger dimensions than the columns of the mixing matrix. In fact, we ran into a problem unrelated to our testing method, which is that just storing the independent components in memory takes a lot of space and ultimately seems to limit the dimensions we can use^6^. Thus, the poorer performance is rather related to the size of the data being analyzed and not the testing method itself.

Based on the computed graphs of memory and CPU time consumption, it is possible to extrapolate and approximate what amount of computational resources are sufficient for a given *n* = *r*, knowing that our computer was sufficient for the cases mentioned above. The results are of course a very rough approximation since they depend on implementation details, and because the number of values of *n* = *r* we used was limited. We set the target at *n* = *r* = 512 which would correspond to rather long recordings of hundreds of subjects collected in a database. Simple linear extrapolations indicate 7 GB of memory is sufficient for testing the mixing matrix, and 150 GB for testing the components. Thus, while testing the mixing matrix is not a problem even for many computer systems at the time of this writing, the testing of components is more challenging. The number of voxels might also be larger than the 10,000 we used above, which would further increase the memory requirements. Likewise, we can extrapolate the computation times needed in the case *n* = *r* = 512: whether testing the mixing matrix or components, the computations would take some 30 h on our modest computer system, so the computation time is really not the bottleneck here.

### 4.4 Experiments on real fMRI data

Table 1 shows the number of clusters found, the average number of components per cluster, and the total number of components clustered for the 5 different parameter settings.

**Table 1:** **Results on real resting-state fMRI data**.

PCA dim	*α*	Linkage strategy	Clusters found	Avg. comps per cluster	# Comps clustered	% Comps clustered
48	0.05	Single	25	6.92	173	36.1
48	0.05	Complete	36	4.14	149	31.0
48	0.01	Complete	34	3.88	132	27.5
75	0.05	Complete	93	3.92	365	48.7
25	0.05	Complete	18	4.22	76	30.4

*The testing and clustering method was applied by varying the PCA dimension, the false positive rate *α* = *α*_FP_ = *α*_FD_, and the linkage strategy during hierarchical clustering*.

Typically, the method assigned a bit more than 30% of the independent components to one of the clusters. Interestingly, the percentage of components clustered was much higher, almost 50%, when the PCA dimension was increased, which indicates that 48 principal components may not be enough. A larger PCA dimension may be necessary to be able to find more corresponding components in different subjects.

We also see the well-known phenomenon where complete-linkage clustering leads to smaller clusters, but produces more of them. We found that single-linkage in fact produced clusters which were sometimes quite heterogeneous (results not shown), so complete-linkage may be preferred on this data. On the other hand, the total number of component clustered is smaller for complete-linkage, because it requires all the connections from the cluster to be significant, which is a more conservative criterion.

Obviously, a smaller *α* leads to fewer clusters and fewer components in the clusters, but the difference between 0.01 and 0.05 is rather small.

**Figure 5:**
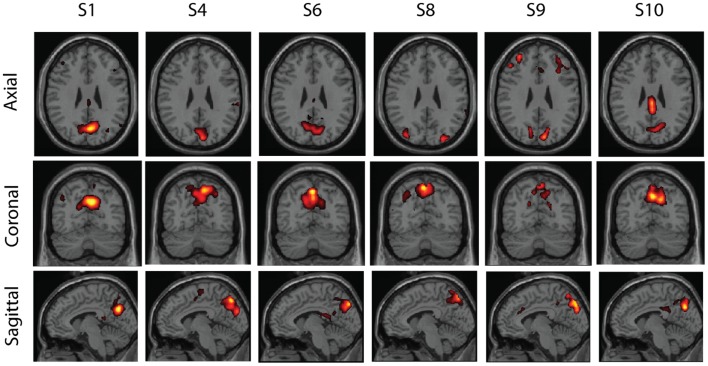
**One cluster found in real resting-state fMRI data**. The component was found in sufficiently similar form in six subjects (out of 10). The cluster seems to correspond to a part of the default-mode network, centered in the precuneus.

**Figure 6:**
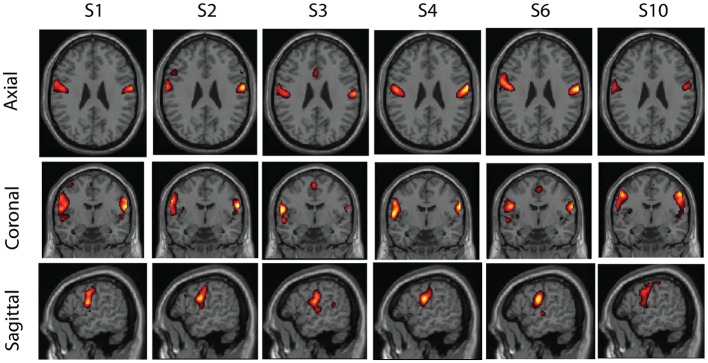
**A second cluster found in real resting-state fMRI data**. This cluster also has components from six subjects, and seems to correspond to bilateral motor areas.

**Figure 7:**
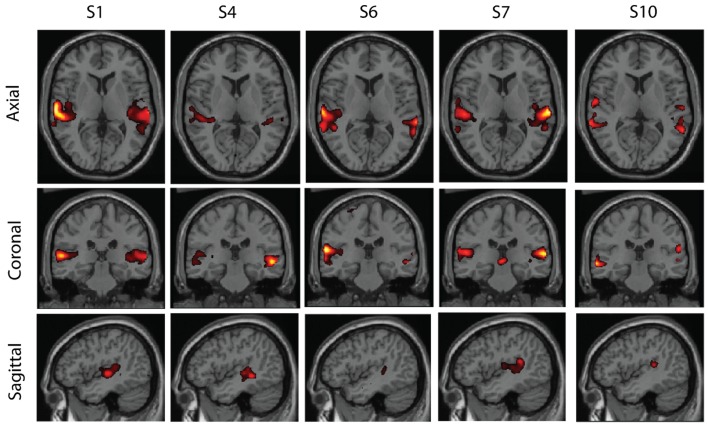
**A third cluster found in real resting-state fMRI data**. The cluster has components from five subjects and seems to correspond to auditory areas.

Some examples of the clusters are shown in Figures 5–7. These were obtained in the basic setting where PCA dimension was 48, complete-linkage was used, and the *α* levels were 0.05. The first cluster in Figure 5 seems to consist of a part of the default-mode network, the second in Figure 6 seems to be a motor network, and the third in Figure 7 is an auditory area. The clusters contain components from 5 to 6 subjects. The clusters were manually selected to reflect some well-known resting-state networks.

## 5 Discussion

In this paper, we extended our previous work (Hyvärinen, 2011) on testing the ICA mixing matrix to testing the values of the independent component patterns. An important application for the present method is spatial ICA of fMRI, especially in resting-state. We proposed an empirical model of the null distribution, whose parameters can be directly estimated from the observed data. We further proposed improvements to the general framework, applicable to both our present and earlier testing methods; they simplify the theory of FDR computation, reduce the computational requirements, and provide alternative clustering strategies.

While the idea of doing a separate ICA on each subject, followed by clustering, is not new (Esposito et al., 2005), the method proposed here is, to the best of our knowledge, the first one which associates statistically principled p-values to each cluster. Thus, the method indicates which clusters should be included in any further analysis and which should be discarded, with a principled computation of the similarity thresholds.

Matlab code for computing the tests proposed in this paper is freely available at www.cs.helsinki.fi/u/ahyvarin/code/isctest/.

### 5.1 Utility in fMRI analysis

Results on real fMRI group data showed reasonable clustering of components to clusters similar to well-known resting-state ICA networks.

Some well-known networks may also be split into more than one cluster. The splitting may be due to individual variability of the spatial patterns. The probability of such splitting depends on the *α* value as well as the clustering strategy. It is well-known in the theory of hierarchical clustering that complete-linkage tends to create clusters which are smaller, but at the same gives more clusters than single-linkage.

Another factor which has a strong effect on the splitting of clusters, independently of individual variability or our testing method, is the PCA dimension. Its effect was systematically investigated by Abou-Elseoud et al. (2010), who found that with a PCA dimension of 10, a single default-mode network is found. For larger dimensions, it is often split into at least two components, and at a PCA dimension of 50 (very close to 48 used above), even four components.

These factors should largely explain why, for example, the cluster related to the default-mode network in Figure 5 contained only the precuneus and only from six subjects. In fact, other clusters containing parts of the default-mode network were found as well (but not shown). Increasing the false positive and false discovery rates and using single-linkage would prevent such splitting of clusters to some degree but at the risk of too permissive clustering of components which may not be related enough. Even so, simply due to the effect of our relatively large PCA dimension, it seems unlikely that we could capture the whole default-mode network in a single cluster. This problem might possibly be alleviated by estimating the number of independent components separately for each subject (Beckmann and Smith, 2004), but determining the dimension automatically is not easy as discussed by Abou-Elseoud et al. (2010). Another factor that might be relevant is the large age range of the subjects; age was shown to change resting-state networks by Dosenbach et al. (2010), so our group might have particularly small inter-subject consistency.

Our method does not by any means discard artifacts, which sometimes form consistent clusters as well, although we only showed resting-state networks above. In fact, the testing method does not seem to contain anything which would prefer components of real brain activity over any kind of artifacts (whether physiological or technical). ICA is well-known to find many artifacts, and the present method just considers all components on an equal footing. Of course, it might be possible that some artifacts are either more or less consistent than brain activity, but we are not aware of results showing any such systematic differences. An automatic method for detecting which components are artifacts was proposed by Tohka et al. (2008).

### 5.2 Relationship to other methods

Related testing methods were proposed by Perlbarg et al. (2008); Varoquaux et al. (2010); Schöpf et al. (2010). Schöpf et al. (2010) used principled statistical methods based on GLM to quantify the similarities between the components, and to rank them in order of consistency. Perlbarg et al. (2008) applied bootstrapping to test the consistency of inter-subject consistency grouping, but the grouping itself used similarity thresholds which were not statistically principled. Varoquaux et al. (2010) applied the idea of random orthogonal rotations like Hyvärinen (2011), but not over different subjects. While all the work cited above used statistical methods to quantify the similarity and/or significance of components, none of them directly addressed the problem we are concerned with: obtaining principled p-values for each component.

An alternative utility of single subject ICA was proposed by Yang et al. (2012), who did ICA on individual subjects and then clustered the *subjects* instead of components based on the inter-subject consistencies of the components.

### 5.3 Relationship to our previous testing method

Our empirical approach introduced above is closely related to the original testing method by Hyvärinen (2011). Thus, we need to understand the differences between the two methods.

#### 5.3.1 Which testing method should be applied?

First we would like to clarify when the different testing methods should be applied. While both methods are applicable in the myriad of application fields where ICA can be applied, we consider only brain imaging data in the following discussion.

The choice of testing method really depends on the combination of two factors: whether we do temporal or spatial ICA, and what the experimental paradigm is. (The imaging modality *per* *se* plays a smaller role here, but it affects the choice of temporal vs. spatial ICA.) The discussion of whether temporal or spatial ICA is to be performed for a given data set is a completely separate one (see, e.g., Calhoun et al., 2009). First one should decide which type of ICA is the right one, and only then choose the testing method.

ICA is typically applied on data on which we can only perform one of the tests. This is the case when the data come from a resting-state study, from a study where the responses are induced but not time-locked to stimuli and hence not correlated (e.g., event-related suppression in EEG/MEG), or any non-resting study with no systematically evoked responses. For such data, we can assume the spatial patterns to be similar, but not the time courses.

Basically, in the case of *temporal* ICA for such data, we would typically assume that the mixing matrix is approximately the same over subjects, since the mixing matrix gives the spatial patterns of activity. This is the case whether we analyze EEG, MEG, or fMRI. (Temporal ICA on fMRI is very rare, however.) So, we should test the mixing matrix using the method by Hyvärinen (2011). Testing the independent component patterns would not be meaningful since they correspond to the activity time courses which cannot be assumed to be correlated here.

Next we consider the cases where the data comes from *spatial* ICA, and from the experimental paradigms mentioned above (resting-state or similar). Then, it is typically the independent components (**S***_k_*) which are approximately the same over subjects, since they correspond to the spatial patterns. So, we should test the independent components themselves, using the method in this paper. Testing the mixing matrix would not meaningful here, since again, the time courses cannot be assumed to be correlated over subjects in the above-mentioned cases. In particular, the popular spatial ICA of resting-state fMRI needs our new testing method proposed in this paper, and cannot be done with our previous method.

However, in some cases it may be possible to apply either of the two tests. This is the case when the data comes from an evoked response study in which the responses for different subjects are similar enough in the sense of being strongly correlated. This is because then both the spatial patterns and the time courses can be tested for inter-subject consistency. The choice of testing method then depends on which of the inter-subject consistencies is stronger, or more interesting from the viewpoint of the study. For example, in an evoked response study with fMRI, after applying spatial ICA, it may be particularly interesting to apply the testing on the mixing matrix to see if the responses themselves (and not just the spatial patterns) have inter-subject consistency.

#### 5.3.2 Similarity measures and effective dimensions

Next, we consider the connections between our two testing methods from the viewpoint of the theory.

In both testing methods, we compute similarities between the components. One important difference is that Hyvärinen (2011) used a weighted Mahalanobis similarity, whereas here we use simple correlations. Related to this, it was assumed by Hyvärinen (2011) that the covariances of the subjects are equal. These two assumptions made it possible to analytically derive the null distribution in Hyvärinen (2011), while here we used an empirical model of the null distribution.

However, these two differences may not be as large as they seem. In fact, let us first consider what happens if we use our empirical model of the similarities when testing the mixing matrix. Suppose that we are testing the similarities of mixing matrices like Hyvärinen (2011), and the covariances of the subjects are equal. Theorem 1 by Hyvärinen (2011) shows that the Mahalanobis similarity matrix is a random orthogonal matrix under the null hypothesis. Thus, its sum of squares equals the data dimension, and the estimate *ñ* of the effective dimension we would get from equation (10) is equal to the data dimension. This means that the empirical model of the null distribution would be equal to the one used by Hyvärinen (2011). Thus, if we use our empirical approach to modeling the similarity matrix in testing the mixing matrix, we recover exactly the same null distribution which was analytically derived by Hyvärinen (2011), provided that the assumption of equal covariances holds. In this sense, the present empirical method is a generalization of our earlier method.

On the other hand, one may ask if we could or should we use the Mahalanobis distance in testing independent components like in this paper. This does not seem necessary because if we adapt the assumptions in Hyvärinen (2011) to the present case, we in fact obtain the simple similarity measure used here. Since the **S***_k_* have orthogonal rows of unit variance, as assumed above, the weighting matrix in the Mahalanobis similarity is equal to identity in the subspace spanned by the rows of **S***_k_*. Thus, any weighting in the distance measure would disappear. In this sense, our present method is rather a special case of the framework by Hyvärinen (2011).

The two points above show that the apparent differences in the definition of the similarities and effective dimensions are much smaller than it seems. Rather, one might see our earlier method and the method proposed here as two instances of the same method, adapted to the parameters inherent to the testing of the mixing matrix or the independent components, respectively.

There is one practical difference, however. In the empirical method proposed here, we do not re-estimate the effective dimension after deflating away components, as was done by Hyvärinen (2011). This makes the present test less conservative. The effect of such re-estimation of the dimension would probably be much smaller here because the effective dimension *ñ* is higher (typically of the order of hundreds), so reducing it by the number which is of the same order as the number of components (typically not more than one hundred) as in our earlier method would not change much. Moreover, it may not be necessary even on theoretical grounds because it is closely related to the assumption of equal covariances. If the covariances are not equal over subjects, the vectors are much less constrained and such reduction of degrees of freedom does not happen. There is some the risk that this makes the test too permissive and creates false positives. However, according to the simulations presented, this does not seem to be the case in reasonably realistic scenarios.

#### 5.3.3 Modeling of the independent components

Any modeling of the independent components using a distribution *p_s_* was not necessary in our earlier method (Hyvärinen, 2011), since the analysis was exclusively concentrated on the estimated mixing matrices. Introduction of *p_s_* in this paper basically means that we admit that there is some additional source of uncertainty. Taking the empirical approach means that we further admit we cannot explicitly model the independent components, i.e., *p_s_*, because of their complexity. This uncertainty is then implicitly modeled by fitting the parameter *ñ* (or *β*) to the data. Thus, the present method is a generalization of our earlier method in this sense as well: we allow for more uncertainty under H_0_, and adapt to it empirically.

In addition to modeling individual differences, another practical meaning of *p_s_* is modeling measurement noise. Any measurement noise is still present in the independent components, and its effect on the similarities has to be modeled. This is in contrast to similarities of columns of the mixing matrix: since measurement noise is basically averaged out in the estimates of the mixing matrix, it can largely be ignored.

### 5.4 Applicability to different ICA algorithms

In the simulations above, we used FastICA. However, no part of the derivation of the testing method assumed that we would use FastICA instead of other ICA algorithms. The only assumption related to ICA estimation was that the estimation is divided into two parts: whitening and finding an orthogonal mixing matrix. Most ICA and blind source separation algorithms, including SOBI (Belouchrani et al., 1997), AMUSE (Tong et al., 1991), and JADE (Cardoso and Souloumiac, 1993), use the same division of estimation into two stages, so our method is just as applicable to them as it is for FastICA.

The notable exception among the ICA algorithms is the infomax algorithm (Bell and Sejnowski, 1995; Amari et al., 1996), which does not require such a division into two stages. However, most implementations of the infomax algorithm do use a preliminary whitening to speed up the algorithm, effectively using two estimation stages as above. Yet, there is usually no constraint of orthogonality of the mixing matrix in the infomax algorithm. This means that our method may not be fully justified for the infomax algorithm. On the other hand, by the definition of the ICA model, even the infomax algorithm should asymptotically give an orthogonal mixing matrix for whitened data, under the theoretical assumption that the ICA model holds. Thus, the assumptions of our method are approximately correct even for the infomax algorithm. Whether this approximation is good enough in practice is an empirical question that we leave for future research.

### 5.5 Computational implementation details

We proposed a simple way of speeding up computation by storing only the maximal similarities in memory. This is not exactly equivalent to using all of them as in our earlier method (Hyvärinen, 2011) but the difference is likely to be very small. This improvement can be used with the testing method in Hyvärinen (2011) as well, and, indeed, with many related methods (Himberg et al., 2004; Esposito et al., 2005).

In Simulation 3, the bottleneck of the computations was seen to be in the large size of the spatial patterns themselves, which we stored in the memory. Thus, the bottleneck is essentially in the database implementation, and not in our testing method *per* *se*. A further computational improvement would presumably be obtained if we didn’t try to hold all the independent components in the memory at the same time. This would require some relatively simple programing solutions in which only part of the ICA outputs are loaded into memory at the same time for computation of the similarities. Such methods might be quite slow because of the disk access needed but they would expand the possibilities of the testing method. However, we leave such database technicalities for future research.

### 5.6 Group ICA and testing

The method developed here can also be viewed as a method for group ICA, if the datasets come from different subjects, as originally proposed by Esposito et al. (2005) and further developed, among others, by Wang and Peterson (2008) and Schöpf et al. (2010). The approach is quite different from conventional group ICA methods (Calhoun et al., 2009) in which the primary goal is to obtain a set of group-average components which characterize the whole group. Such a set of average components can then be used to compute the corresponding components in each subject. Malinen et al. (2010) originally applied such a method (GIFT) on the data we re-analyze here, so comparing the present results to theirs will give a general idea on the differences and commonalities of the two analyses.

Estimating group-level component has been further advanced by Beckmann and Smith (2005), whose tensorial ICA method allows some inter-subject variability in both the independent components and the mixing matrix; however, tensorial ICA assumes the component time courses to be similar for all the subjects in whom the component is present, so it is hardly applicable to spatial ICA of resting-state fMRI. Guo and Pagnoni (2008) further proposed a principled expectation-maximization approach for estimating group components.

A possible problem with estimating group-level components is that there is no guarantee that the component “exists” in each subject, since the subject-wise components are computed by simple formulas without any checking that the obtained component matches. the data of the subject in question. The question of whether the components obtained by group-level ICA are present in single subjects was considered by Erhardt et al. (2011) and Allen et al. (2012). The main advantage of computing a separate ICA for each subject is that there is more certainty that the subject-wise components really correspond to the statistical properties of the subject (Esposito et al., 2005).

On the other hand, computing a separate ICA for each subject may have the disadvantage that the estimation of the components does not use all the information available, in particular the information that the components are likely to be similar in the different subjects. In fact, in the fMRI results above, the components were hardly ever found in more than half of the subjects. While this may be an accurate description of the underlying individual differences in neurophysiology and anatomy, it is also possible that this is a conservative estimate. For example, due to the algorithmic randomness of ICA algorithms (Himberg et al., 2004), the components obtained are just a subset of the larger set of all the possible components. In ICA estimation, there is thus an aspect of random sampling from this pool of components, which reduces the number of matches that can be found by a clustering algorithm like the one proposed here.

A possible compromise would be to use a framework similar to Varoquaux et al. (2011), which develops an explicit model of the components, and in particular their individual differences. This is an interesting direction for future research. However, such models cannot be straightforwardly used for the testing of the components because the components are not estimated independently in different subjects. Another important question for future research is how comparison between groups can be done in the present testing framework. Any methods applicable for the original framework by Esposito et al. (2005) are likely to be applicable for our method as well.

## Conflict of Interest Statement

The authors declare that the research was conducted in the absence of any commercial or financial relationships that could be construed as a potential conflict of interest.

## Footnotes

^1^In our implementation, we handle ties inherent in median calculation by requiring that more than one half of the connections must be significant.

^2^For a grid of this size, the effective dimension is at most 625, and typically of the order of hundreds. In the fMRI experiments reported below, the *ñ* was in the range of 100 … 500, depending mainly on the PCA dimension.

^3^While we could have created the inconsistent patterns to contain activity blobs as well, this would have created the problem that some of the supposedly inconsistent patterns would have been strongly correlated by chance. Then, the validation of the method would have failed since the distinction between consistent and inconsistent patterns would not have been well-defined.

^4^http://www.fil.ion.ucl.ac.uk/spm/

^5^To characterize the significance of the improvement on a more anecdotal basis, let us point out that in Hyvärinen (2011) we could do *n* = *r* = 64 on an ordinary computer (the very same computer as in the current experiments), while *n* = *r* = 128 was impossible because we ran out of memory. With the new optimized method which stores maximal connections only, we were able to compute *n* = *r* = 128 as well as *n* = *r* = 256. The case *n* = *r* = 512 was not feasible because the system ran out of memory.

^6^Again, considering the performance of our particular computer system: We were able to do only *n* = *r* = 64 since simply storing the independent components in memory took 37% of it.
